# Evaluation of Different Methods for Cultivating *Gluconacetobacter hansenii* for Bacterial Cellulose and Montmorillonite Biocomposite Production: Wound-Dressing Applications

**DOI:** 10.3390/polym12020267

**Published:** 2020-01-26

**Authors:** Katharine Valéria Saraiva Hodel, Larissa Moraes dos Santos Fonseca, Isa Moreira da Silva Santos, Jamile Costa Cerqueira, Raimundo Evangelista dos Santos-Júnior, Silmar Baptista Nunes, Josiane Dantas Viana Barbosa, Bruna Aparecida Souza Machado

**Affiliations:** 1 University Center SENAI CIMATEC, National Service of Industrial Learning, Laboratory of Pharmaceutical’s Formulations, Health Institute of Technologies (ITS CIMATEC), Salvador 41650-010, Brazil; katharine.hodel@fbter.org.br (K.V.S.H.); larissa.fonseca@fbter.org.br (L.M.d.S.F.); jamilecosta@msn.com (J.C.C.); josianedantas@fieb.org.br (J.D.V.B.); 2 University Center SENAI CIMATEC, National Service of Industrial Learning, Salvador 41650-010, Brazil; isamoreira@outlook.com.br (I.M.d.S.S.); rjs.evangelista@gmail.com (R.E.d.S.-J.); silmar@fieb.org.br (S.B.N.)

**Keywords:** bacterial cellulose, biocomposites, montmorillonite, wound dressing, ex situ production, carbon sources, *Gluconacetobacter hansenii*

## Abstract

Bacterial cellulose (BC) has received considerable attention due to its unique properties, including an ultrafine network structure with high purity, mechanical strength, inherent biodegradability, biocompatibility, high water-holding capacity and high crystallinity. These properties allow BC to be used in biomedical and industrial applications, such as medical product. This research investigated the production of BC by *Gluconacetobacter hansenii* ATCC 23769 using different carbon sources (glucose, mannitol, sucrose and xylose) at two different concentrations (25 and 50 g∙L^−1^). The BC produced was used to develop a biocomposite with montmorillonite (MMT), a clay mineral that possesses interesting characteristics for enhancing BC physical-chemical properties, at 0.5, 1, 2 and 3% concentrations. The resulting biocomposites were characterized in terms of their physical and barrier properties, morphologies, water-uptake capacities, and thermal stabilities. Our results show that bacteria presented higher BC yields in media with higher glucose concentrations (50 g∙L^−1^) after a 14-day incubation period. Additionally, the incorporation of MMT significantly improved the mechanical and thermal properties of the BC membranes. The degradation temperature of the composites was extended, and a decrease in the water holding capacity (WHC) and an improvement in the water release rate (WRR) were noted. Determining a cost-effective medium for the production of BC and the characterization of the produced composites are extremely important for the biomedical applications of BC, such as in wound dressing materials.

## 1. Introduction

Bacterial cellulose (BC) is a natural biopolymer that is achieving worldwide attention because of its simple and feasible production technology. BC has the same chemical formula of plant cellulose (C_6_H_10_O_5_)n with β-D-glucopyranose units interlinked by intermolecular hydrogen bonds [1,2]. However, unlike plant cellulose, BC has an ultrafine network structure with high porosity and thinner fibrils (nanometers sized). Moreover, BC possesses distinguished features, such as high purity (free of lignin, hemicellulose, and pectin), high degree of polymerization, high crystallinity, high water content, high mechanical stability and great chemical modifying ability [3,4,5,6]. These unique characteristics make BC a promising alternative to plant-derived cellulose for several biomedical applications being widely studied, including wound dressing materials [7,8,9].

Development and application of natural polymer-based wound dressing has been described in various studies and patents in recent years [10,11,12,13,14]. Traditional dressings, such as gauze, lint, plasters, bandages (natural or synthetic) and cotton wool, have limitations in epidermal or skin damage or burns, as they fail to prevent the growth of microbial infections and provide a moist environment for healing [15]. An ideal dressing not only covers and protects the affected area but also optimizes the wound environment to facilitate healing [9,15]. Biomedical applications of BC have received considerable attention, for example, in wound dressing [16,17], scaffold implants [6] skin and bone tissue engineering [18] and delivery systems of drug [19,20].

The moisture retaining property of BC helps to maintain homeostasis, aid healing, reduce pain, minimize scar formation and increase the epithelialization rate; thus, BC represents a great alternative to traditional dressings [9,15]. Additionally, BC constitutes a great matrix for the synthesis of functional composites due to its huge nanoporous area and the presence of oxygen atoms rich in electrons [15,21,22,23]. Therefore, some researchers have investigated BC-reinforced polymer composites with different materials, including conducting materials [24], graphene oxide [20], carbon nanotubes [25], ceramics [26], and biopolymers [27], for various biotechnological applications.

In the past few years, polymer nanocomposites with numerous clays have been extensively investigated mainly due to their cost effectiveness and possible applicability in various areas, including structural, electronics, and biomedical industries [28,29,30]. Montmorillonite (MMT) is a surface-reactive hydroxyl-layered aluminum silicate clay mineral that forms microscopic platinum crystals [31]. MMT is one of the most widely used medicinal clays and has interesting properties for wound dressing applications, such as wound healing and antimicrobial properties, cleansing, skin protection, blood clotting and hemorrhage control in trauma patients [21,32,33]. As dressing material, MMT has been demonstrated by previous studies to be able to enhance the BC wound healing properties by adding to its antibacterial properties and improving its physical-mechanical and thermal properties [21,32,34,35].

Among cellulose producing bacteria, the most effective species are from the *Gluconacetobacter* genus (also named as *Acetobacter*) [36,37]. When these species are grown in a laboratory under static conditions, cellulose forms as a swollen membrane in the air-water interface, which increases in thickness if cultivation time increases [37]. There are several reports of the use of different media and carbon sources in the literature [38,39,40,41]. However, the low yield of the static production process and the high production cost of BC have restricted the commercial efficacy of BC. Finding an optimal culture medium and appropriate growth conditions that facilitate higher yields of cellulose would aid in the viability of this technology in an industrial situation.

Therefore, in this study, we evaluated BC membrane production by *Gluconacetobacter hansenii* ATCC 23769 under static conditions using different carbon sources (glucose, sucrose, maltose, and xylitol) at different concentrations. The BC produced was used to synthesize BC-MMT biocomposites with 0.5, 1, and 2% MMT to produce a biomaterial with enhanced physical-mechanical properties for biomedical field applications as a dressing material. The properties of the BC-MMT composites, including mechanical strength, thermal stability, water holding capacity (WHC), and water release rate (WRR), were investigated to determine their suitability for applications in various fields. The results demonstrated that the use of a clay mineral such as MMT improves the physical-mechanical properties of BC, making it able to be used as wound dressing. These findings elucidate BC-MMT composites as a promising alternative for various medical treatments such as wound healing and repair of damaged tissue.

## 2. Materials and Methods

**Figure 1:**
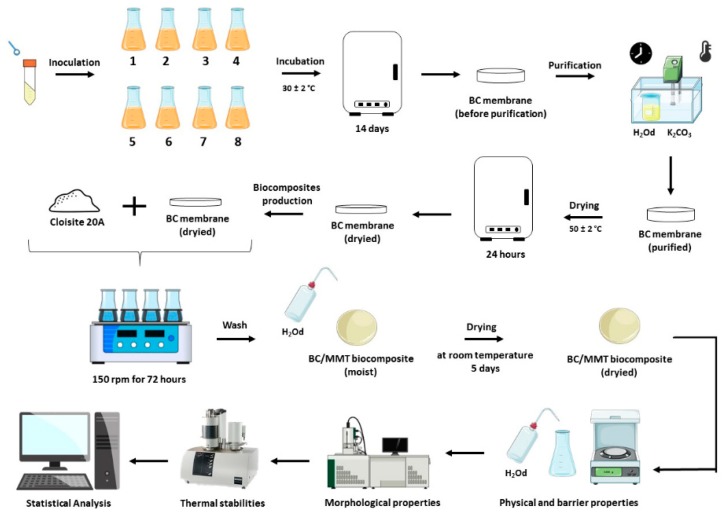
Complete methodology used for BC and BC-MMT biocomposite production and characterization.

Figure 1 illustrates the complete methodology used in the production of the BC and BC-MMT biocomposites. BC was produced by inoculating *G. hansenii* ATCC 23769 in modified HS-media until BC membrane production was observed (14 days). The BC membrane was submitted to purification processes and dried for 24 h, then the pure and dried BC membrane was exposed to Closite 20A (MMT) to form the BC-MMT biocomposite moisture. The BC-MMT moisture was dried at room temperature for 5 days and characterized in terms of their physical and barrier properties, morphologies, and thermal stabilities.

### 2.1. Bacterial Strain, Culture Media and Production Kinetics

The *G. hansenii* strain (ATCC 23769), obtained from the Tropical Culture Collection (CCT)—André Tosello Foundation, was used in the static fermentation process to obtain BC. The culture was grown in modified Hestrin-Schramm (HS) media containing 25 or 50 g∙L^−1^ carbon source (glucose, mannitol, sucrose, and xylose), 5 g∙L^−1^ yeast extract, 3 g∙L^−1^ peptone, and 2 g∙L^−1^ KH_2_PO_4_ (Table 1). All the culture media were sterilized (121 °C, 15 min) and incubated at 30 °C for 14 days. BC production kinetics were evaluated (OD_600_) until BC production was observed.

### 2.2. BC Purification, Yield Calculation and Dry Film Formation

BC membranes were purified by alkaline treatment with potassium carbonate (K_2_CO_3_). The membranes were washed twice with distilled water at 80 °C for 1 h to remove impurities from the culture medium. Then, the membranes were treated with 0.3 mol∙L^−1^ K_2_CO_3_ aqueous solution at 80 °C for 1 h and washed with distilled water until a neutral pH was obtained. The purified BC membranes were dried at 50 °C for 24 h and weighed before and after drying.

### 2.3. Preparation of the BC-MMT Biocomposites

The BC-MMT biocomposites were produced as previously described by Perotti et al. [19]. MMT (Cloisite 20A) was vigorously crushed in a crusher (Turratec TE-102) for 10 min to produce a homogeneous suspension with smaller particles. The BC-MMT nanocomposites were prepared by mixing BC and MMT suspensions in a mechanical shaker at 150 rpm for 72 h. The biocomposites were removed from the beakers, washed with deionized water and dried at room temperature for 5 days. The MMT proportions in the BC-MMT composites were 0.5, 1.0 and 2.0%; the samples were labeled as bacterial cellulose BC (sample without MMT), BC–MMT0.5, BC–MMT1, and BC–MMT2, respectively (Table 2).

### 2.4. BC and BC-MMT Biocomposite Characterization

#### 2.4.1. Water Activity and Water Absorption Capacity

The measurements of water activity (a_w_) were performed using a Decagon (Novasina, Lab Master a_w_, Switzerland) at a temperature of 25 °C. The equipment software applied an equation to evaluate the “actual balance” [a_w_ = moisture in the balance sheet = actual balance (%)/100] [42]. The analyses were performed in triplicate.

The water absorption capacity (WAC) of the membranes (biocomposites) was assessed using 1.5-cm-diameter samples. The initial dry mass was obtained after drying at 50 °C ± 2 °C for 2 h. The specimens were immersed in 50 mL of distilled water for 2, 24, 48, 66 and 72 h. After the predetermined intervals, the specimens were removed, and the excess water was absorbed on filter paper for 1 min. The hydrated films were reassessed. The rehydration rate was calculated according to Equation (1):

WAC = (water mass removed along drying (g))/(dried mass of the sample (g))(1)

#### 2.4.2. Grammage and Thickness

Grammage was determined according to Almeida et al. [43]. A 2-cm^2^ area of each film was weighed on an analytical balance to determine sample mass. The weight was calculated as the ratio between the mass found and the area. The analyses were performed in triplicate. The thickness of the films was evaluated by analyzing the mean thickness of ten measurements at random positions of each respective formulation using a flat point digital micrometer (Digimess; Ip40, São Paulo, Brazil; 0.001-mm resolution).

#### 2.4.3. Water Holding Capacity and Water Release Rate

The ability of the films to conserve water was evaluated by the water holding capacity (WHC) according to Schrecker and Gostomski with adaptations [44]. The membranes were cut into circular specimens (2 cm² in diameter), and their initial dry weight (Pd) was measured. Then, the membranes were immersed in deionized water for 48 h. The swollen membranes were then rapidly dried with filter paper to remove excess surface water and placed in open Petri dishes at room temperature. The membranes were removed from the plates every 24 h to be weighed (Pm) with a total analysis time of 96 h. The WHC was defined by Pm divided by Pd. To determine the water release rate (WRR), the wet masses of the samples were measured following continuous weighing of the samples stored under ambient conditions at different time intervals until a constant mass was obtained. The mass obtained at different time intervals was expressed as a function of time [21]. The WHC and WRR analyses were performed in triplicate.

#### 2.4.4. Thermogravimetric Analysis (TGA)

Thermogravimetric analysis (TGA) was carried out on a Q50 thermogravimetric analyzer (TA instruments, Bavaria, Germany). For this analysis, approximately 8 mg of each sample was placed in a platinum crucible at a heating rate of 10 °C min^−1^ from 25 °C to 1000 °C under a nitrogen flow (30 mL min^−1^), according to Machado et al. [45]. Differential thermogravimetry (DTG) curves were calculated from the TGA results.

#### 2.4.5. Scanning Electron Microscopy (SEM)

Scanning electron microscopy (SEM) (BX-51; Olympus, Tokyo, Japan) was used to study the surface morphology and elements of the nanocomposites. The samples were manually fixed using tweezers (PELCO1 Tweezers) with aluminum metal surfaces covered with carbon double-sided tape, called stubs, according to that previously described by Machado et al. [46]. Due to the need for interaction of the electron beam with the samples, the method was performed by coating deposition of metallic gold ions (sputtering). The samples were metalized in gold in a “Sputter oater” from Balzers (SCD 50; BAL-TEC, Grand Island, NY, USA). Then, the stubs containing the metallic samples were stored in plastic boxes (storage boxes) and double sealed with Parafilm (PARAFILM1 M) to prevent moisture absorption. The samples were analyzed at different magnifications (Voltage 12 kV, Working Distance 12 mm, Spot size 44, Vacuum Mode HV).

### 2.5. Statistical Analysis

The results are expressed as the mean ± standard deviation (n = 3). For the statistical analysis, the program Statistica 6.0 from StatSoft (Tulsa, OK, USA) was used. Analysis of variance and Tukey’s test were used to identify significant differences between the means (*p* < 0.05) of the investigated parameters.

### 2.6. Experimental Program

Table 3 shows the experimental program of the study, with the analyzed specimens, the tests performed and their respective parameters.

## 3. Results and Discussion

### 3.1. Effect of Different Carbon Sources on Cellulose Yields

**Figure 2:**
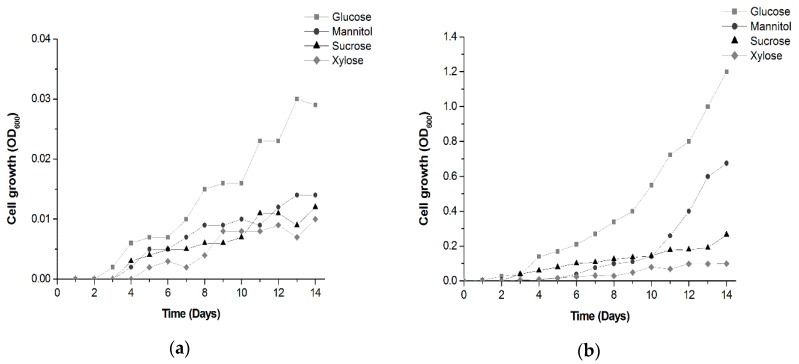
Effect of different carbon sources at 25 g∙L^−1^ (**a**) and 50 g∙L^−1^ (**b**) concentrations on bacterial growth over time. BC was fermented by static culture for a 14-day period.

Due to its unique properties, BC has drawn attention from industries exploring novel strategies for improving BC production. Unfortunately, large-scale production and industrialization of BC are limited due to the low-cost effectiveness of cultivation in traditional synthetic media and static cultures. As a result, numerous efforts have been made to develop new methods for BC production. As described in different studies, BC production can be greatly influenced by a range of variants, such as bacterial strain, reactor type, pH, temperature, aerobic conditions, and nutrient concentration in the culture media [39,40,47,48,49,50]. Among these nutrients, the influence of the carbon sources has been the object of continuous studies [49,50]. The present study highlights the ability of *G. hansenii* ATCC 23769 to metabolize several carbon sources in modified HS media and the effects on cellulose production. The bacteria were incubated for 14 days, which is the time until the first BC production was observed, and cellulose was extracted to examine the yields. Figure 2 shows the bacterial growth over time.

Bacterial growth was observed in all media as expected, since *G. hansenii* is able to assimilate various sugars [37,47,51]. However, better growth (optical density, OD_600_) was observed in media with higher carbon concentrations, especially glucose-containing media (Figure 2b). The results are in agreement with other findings demonstrating that *G. hansenii* exhibits better growth than other *Gluconacetobacter* species in glucose-containing media due to its ability to direct its energy towards cell growth instead of polysaccharide production [52,53].

**Figure 3:**
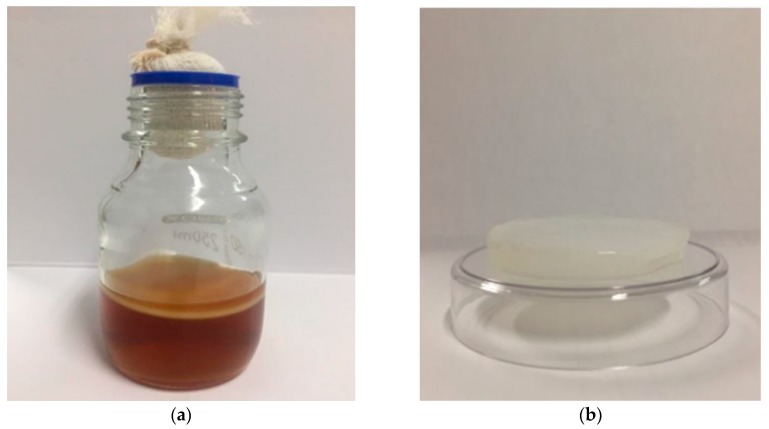
BC produced by *G. hansenii*: (**a**) BC produced in glucose-containing media after a 14-day incubation period; (**b**) pure BC membrane after purification process.

Regarding BC production, BC yields were observed starting on day 14 only in media with glucose in 50 g∙L^−1^ concentration (14.72 g of purified BC) (Figure 3). Media containing mannitol at the same concentration produced BC yields only after 20 days of incubation. BC yields were not observed in media containing sucrose and xylitol as carbon sources at the pre-established concentrations. BC is a biopolymer produced from hexose phosphate generated by phosphorylated exogenous hexoses or indirectly via the pentose cycle and gluconeogenic pathway [54].

Four sugars were used in this study: glucose, mannitol, sucrose, and xylose. Only glucose and mannitol are hexose sugars and produced cellulose yields. The results are consistent with the observations by Rani et al., Molina-Ramírez, and others [41,55,56], who indicated that BC production is affected by carbon source type and concentration (Table 4).

Hutchens et al. [67] evaluated the effects of glucose and mannitol on BC production by *G. hansenii*. In that work, the BC membranes started to appear after 22 days of fermentation. However, these findings show that bacteria produced higher BC yields in mannitol-containing media. Rukka et al. [47] reported similar results comparing media containing glucose, mannitol, and fructose for BC production. In a 7-day *G. xylinus* fermentation period, those findings reveal glucose and mannitol as better carbon sources; however, bacteria produced more BC in mannitol-containing media with 13.5 and 16 mg of cellulose mass production. In a comparative study, Kawano et al. [52] demonstrated that *G. hansenii* presented a twice the growth of *G. xylinum*, while the cellulose synthesis by *G. xylinum* was higher in glucose-containing media; these findings suggest that *G. hansenii* directs its energy towards cell growth, while *G. xylinum* spends its energy on BC production. The difference in BC production using different carbon sources by *Gluconacetobacter* strains is probably because these species have a major number of glucose metabolic pathways to transform glucose into energy for general activities through oxidative metabolism [54]. It is important to mention that there are other factors in addition to carbon sources that influence BC production, such as media pH, nitrogen source, temperature and static or shacking conditions, which are crucial for process optimization [50,55,68].

Our work demonstrates that in modified HS media, *G. hansenii* ATCC 23769 produces higher BC yields in glucose-containing media. An interesting fact is that sucrose and xylose did not lead to any BC production. Since sucrose is a disaccharide composed of two hexose sugars (glucose and fructose), BC synthesis using this sugar requires an additional metabolic step to catalyze the sucrose into glucose and fructose to achieve cellulose production, but greater than 14 days of incubation are required to start producing BC [68]. Molina-Ramírez et al. and Ruka et al. [47,56] have reported low levels of cellulose after an initial incubation period followed by high levels after some days, indicating an increased lag period for cellulose production. In contrast to hexoses, xylose is a poor carbon source for cellulose production by *Gluconacetobacter* species. Ishihara et al. [69] have reported that *G. xylinus* oxides d-xylose to d-xylonic acid, which causes very low pH conditions that are unfavorable for bacterial growth and cellulose production. In this work, xylose would become a utilizable substrate for bacterial strains if xylose-isomerase was added to the medium.

### 3.2. Effect of Different MMT Concentrations on BC Properties

**Figure 4:**
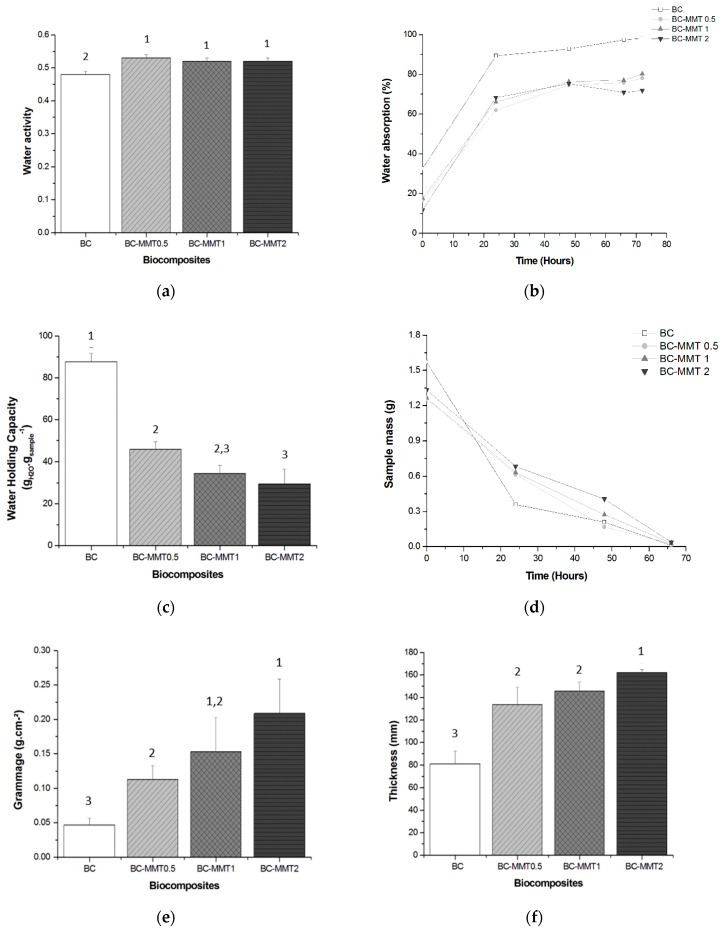
Physical and barrier properties: (**a**) Water activity (a_w_) content; (**b**) Water absorption; (**c**) Water holding capacity (WHC); (**d**) Water release test (WRR); (**e**) Grammage; and (**f**) Thickness of pure BC and BC-MMT biocomposites. No significant difference between values with the same superscript numbers (^1, 2, 3^) in a bar (*p* > 0.05), according to Tukey’s test with 95% confidence.

**Figure 5:**
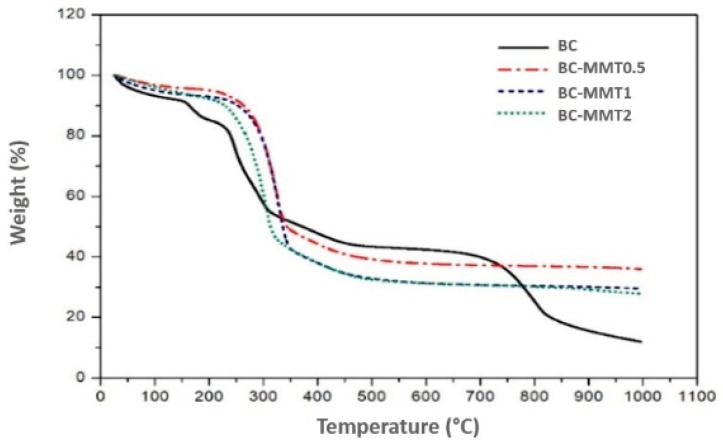
Thermogravimetric analysis (TGA) curves for BC and BC–MMT composites. The composites (BC–MMT1, BC–MMT2 and BC–MMT3) were prepared by impregnation of BC sheets in respective concentrations (0.5, 1 and 2%) of MMT suspensions.

**Figure 6:**
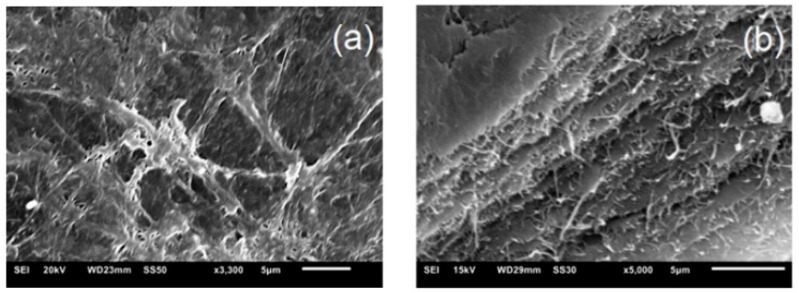
Scanning electron microscopy (SEM) micrographs of the surface and cross-section morphology of pure BC: (**a**) 3300×; (**b**) 5000×.

As previously described, the BC produced was used to develop BC-MMT biocomposites. Flexible membrane formulations of BC-MMT biocomposites were evaluated based on the following features: physical properties (grammage, thickness, a_w_, and water absorption), barrier properties (WHC and WRR), TGA, and morphological properties (SEM) (Table 5, Figure 4, Figure 5 and Figure 6).

Pure BC exhibited an a_w_ value of 0.479 with significant differences (*p* < 0.05) compared with the BC-MMT formulations, which presented higher values ranging from 0.516 to 0.529 (Figure 4a). Moreover, a slight decrease in the a_w_ values was noted in the samples with higher MMT concentrations. The interactions formed in the BC-MMT composite caused an increase in the free water content. In this case, the presence of MMT potentially significantly contributed to this finding. This notion can be justified because if clay is lacking in the formulation, the bonds between the polymers are stronger. Therefore, there is less water available. This fact directly influences the mechanical properties of the materials, since the main functional properties (mechanical and barrier properties) of these hydrophilic materials depend on their water content, as previously described in other studies [42,70]. Several studies have demonstrated that MMT influences the a_w_ in films [71,72,73]; however, Azevedo et al. [73] reported that the free water in whey protein isolate films decreased when MMT is incorporated, and only one sample presented higher a_w_ values compared with other samples.

Currently, advanced approaches to wound healing have attracted great attention for the use of novel types of dressing that provide a moist environment to the wound area [74]. This property encourages fast and effective healing, which is particularly important when dealing with chronic wounds [8,23]. Moreover, a moist environment facilitates the penetration of active substances and potentially allows for an easy and painless dressing change, protecting the newly formed skin from damage. Due to its physical and barrier properties, such as a high WHC and slow WRR, BC has been extensively studied for use as a wound dressing material [15,21,28]. Therefore, in this study, we investigated the WAC, WHC, and WRR of the pure BC and the BC–MMT composites for use in wound dressing applications.

Our results show that the WAC of pure BC increased compared with that of the BC-MMT composites (Figure 4b). The samples of pure BC recovered approximately 90% of their dry mass in 72 h of water immersion, while the hybrid materials recovered approximately 80%. The deposition of MMT particles on the surface and in the matrix of the BC sheets reduces the empty spaces in the BC–MMT composites, which ultimately results in the lower water absorption and lower WHC of the composites. The MMT absorption and penetration inside the BC matrix increases with the MMT concentration, which subsequently results in decreased WHC in the following order, BC > BC–MMT0.5 > BC–MMT1 > BC–MMT2, as shown in Table 5 and Figure 4c. This finding may be explained by the fact that the clay affects the swelling capacity of the material [31]. Any coefficient that reduces the empty spaces between the layers or pores will directly affect this mechanism. Similar results were obtained by other authors who observed a diminution in the water absorbed by nanocomposites made with MMT [48,75,76,77,78]. Taghizadeh et al. [78] investigated the WAC of sodium montmorillonite clay (MMT-Na) content within cellulose blends. In their work, the blends with MMT-Na content exhibited a significantly reduced WAC rate.

In the present study, the WHC of pure BC was 87.729, which decreased to 46.023, 34.550 and 29.514 for BC–MMT0.5, BC–MMT1, and BC–MMT2, respectively (Table 5). Since the hydroxyl groups of MMT can form strong hydrogen bonds with the hydroxyl groups on cellulose, the interactions between the molecules and the cohesiveness of biopolymer matrix are improved. In addition, the water sensitivity is reduced. The clay produces a tortuous pathway, and the length of the path for water uptake is reduced [78]. The presence of MMT particles in the matrix of the BC sheets reduces the empty spaces in the BC–MMT composites, thus reducing water absorption and the WHC of the composites [21]. Moreover, Majeed et al. and Bakar et al. [79,80] have demonstrated that composites with MMT possesses improved mechanical properties tensile and flexural strength, important indicators to manufacture qualified wound-dressing material.

Figure 4d depicts the WRR values through the variation of sample mass stored under ambient conditions over time. Although the initial content of pure BC is much higher than that found in the BC-MMT biocomposites, there was a drastic reduction, resulting in greater than 80% water loss in 48 h. The water content in the pure BC was insignificant after 60 h. However, while the initial water content in all the BC-MMT biocomposites was inferior, demonstrating a low WHC, the WRR is gradual and uniform compared to that of the pure BC. The presence of the MMT particles helps to protect the absorbed water molecules from evaporation, leading to its retention for a longer period inside the BC sheets. Ul-Islam et al. [81] reported similar results by evaluating the WHC and WRR of BC obtained by ex situ modification. In that report, it took approximately 90 h for the complete evaporation of water from the surfaces of composites made with MMT and chitosan. Since the pore size is smaller in these composites (Figure 6), the penetrated water molecules are more tightly sandwiched between the microfibrils.

Figure 4e shows grammage levels of the pure BC and BC-MMT composites. The pure BC presented grammage of 81.250 g cm^−2^, while the BC-MMT composites presented levels between 0.113 and 0.209 g cm^−2^ in BC-MMT0.5, BC-MMT1, and BC-MMT2 with increases of 39, 44 and 49%, respectively. The increase in the MMT concentration influenced the grammage property due to increased clay deposits in the polymer matrix. Grammage is directly related to a film’s mechanical resistance and barrier properties given that higher grammage levels offer higher mechanical resistance [43]. The mean thickness is another important parameter that must be monitored in films for the maintenance of uniformity, and this parameter allows for the validation of the comparisons of their properties. In this work, the pure BC films presented a thickness of 0.047 mm, while the biocomposites presented higher values of 133.667, 145.667 and 162.333 mm for BC-MMT0.5, BC-MMT1, and BC-MMT2, respectively. The influence of the nanoclay particles impregnated in BC grammage and the thickness properties have also been reported by Tunç et al. [77]. That work shows that an increase in the MMT concentration caused an increase in the film thickness and opacity values of methylcellulose films, whereas the increase in the MMT concentration led to a decrease in water adsorption and water solubility.

TGA is a thermal analysis method in which the mass variation of the sample (loss or gain) is determined as a function of temperature and/or time. TGA allows for monitoring the occurrence of various reactions, such as dehydration, oxidation, and degradation. Figure 5 shows the TGA curves (25–1000 °C) of the pure BC and BC-MMT composites. The graphs show two major weight loss zones. Approximately 9% of the mass loss of the pure BC occurs in temperatures between 80 and 120 °C. This finding is in contrast to the BC-MMT materials that presented a mass reduction of only 5%. The weight loss occurring at 80–120 °C is due to the loss of moisture content and interlayer coordinated water molecules [82]. The relatively higher weight loss for the pure BC at this temperature range may be due to its higher WHC compared with the BC–MMT composites as shown in Table 5. In the second phase, degradation of the main cellulose skeleton is initiated at 225 °C in all samples [82]. The maximum weight loss was recorded for all samples during this phase. The weight loss for the pure BC is approximately 45% during this phase (Figure 5). In this phase, the weight of all the BC-MMT composites was lower than that for pure BC. Additionally, with the increased concentration of MMT, the weight loss decreased by approximately 48, 51 and 53% for BC–MMT0.5, BC–MMT1, and BC–MMT2, respectively. Additionally, Figure 5 shows a difference between the degradation of pure BC and BC-MMT composites along this phase. The first difference is noted at 151 °C and was extended to 177 °C for all the formulations.

### 3.3. Effect of Different of MMT on Surface Morphology of BC

Figure 6 shows the SEM images of pure BC and BC-MMT biocomposites. Figure 6a presents a proper fibrous structure of the cellulose produced by *G. hansenii* and its high porosity [59]. The alkaline treatment performed for BC purification influenced in the porous membrane as evidenced by Sulaeva et al. [8]. The membrane sagittal cut (Figure 6b) presents a porous network interconnected via lamellar formation. Porosity is a characteristic that directly influences the adhesion and cellular growth in the dressing material matrix [83].

**Figure 7:**
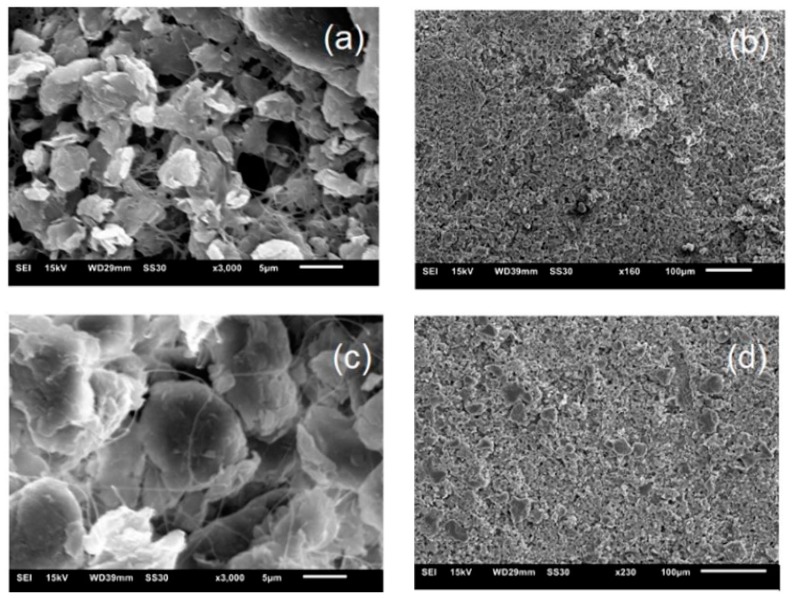
Scanning electron microscopy (SEM) micrographs of the surface and cross section morphology of: (**a**) ×3000 and (**b**) ×160 BC-MMT0.5; (**c**) ×3000 and (**d**) ×230 BC–MMT2.

From the morphological study of the BC samples immersed in the MMT, it was possible to confirm the syntheses of the BC-MMT biocomposites (Figure 7). BC nanofibrils create an expanded superficial area and a high porous matrix that enables the syntheses of a various composites with nanoparticles [60]. The biocomposites SEM micrographs illustrate the three-dimensional arrangement of microfibrils and MMT penetration in BC layers through its porous structure (Figure 7). MMT is impregnated in the BC superficies, and the level of impregnation increases as the MMT concentration increases. The empty spaces between the fibrils in BC-MMT0.5 (Figure 6a) were almost all filled in BC-MMT2 (Figure 7c) due to the increase in particle accumulation. Moreover, the MMT particle size seems to be higher in BC-MMT2. This result could be explained by the particle’s agglomeration in the matrix.

## 4. Conclusions

In this work, BC production by *G. hansenii* ATCC 23769 was evaluated using four different carbon sources at two different concentrations (25 g∙L^−1^ and 50 g∙L^−1^). The BC produced was used to synthesize BC-MMT biocomposites for wound dressing applications. The only production yield was obtained in medium with glucose as a carbon source at 50 g∙L^−1^ concentrations. The BC–MMT composites were prepared through a simple particle impregnation strategy to enhance the physico-mechanical properties of BC. Various analytical techniques, including a_w_, WAC, WHC, WRR, grammage, thickness and TGA, were used to characterize the composites. It was observed a slight increase in the a_w_ values in the samples with higher MMT concentrations, from 0.529 to 0.516. The WAC of pure BC increased compared with that of the BC-MMT composites while the WHC decreased with higher MMT concentrations from 46.023 to 29.514 g_water_ g_sample_^−1^. Regarding grammage, the pure BC presented 81.250 g cm^−2^, while the BC-MMT composites presented levels between 0.113 and 0.209 g cm^−2^. The pure BC films presented a thickness of 0.047 mm, while the biocomposites presented higher values of 133.667, 145.667, and 162.333 mm in higher MMT concentrations. These results show that the physical-mechanical and thermal properties of the composites were significantly improved compared to those of pure BC. Additionally, the BC-MMT composite exhibited improvements in the WRR compared with that of the pure BC. This is an important feature of BC in wound dressing applications. For industrial applications of BC, it is important to find cost-effectiveness conditions. Additionally, the addition of materials, such as MMT, enhances BC properties for biomedical applications. The results reported in this study support the production of BC with good yield while using glucose as carbon source and the composites produced from this polymer with MMT showed interesting results for further analysis of their properties, aiming at their application as wound dressing.

## Figures and Tables

**Table 1:** Media components and concentration.

Medium Components	Culture Medium (%, *w/v*)							
	1	2	3	4	5	6	7	8
Glucose	25.0	50.0	-	-	-	-	-	-
Mannitol	-	-	25.0	50.0	-	-	-	-
Sucrose	-	-	-	-	25.0	50.0	-	-
Xylose	-	-	-	-	-	-	25.0	50.0
Peptone	3.0	3.0	3.0	3.0	3.0	3.0	3.0	3.0
Yeast extract	5.0	5.0	5.0	5.0	5.0	5.0	5.0	5.0
KH_2_PO_4_	2.0	2.0	2.0	2.0	2.0	2.0	2.0	2.0

Legend Table 1: (-) Not added.

**Table 2:** Sample name and montmorillonite (MMT) concentration of biocomposites.

Sample Name	MMT Concentration (%)
BC	0.0
BC-MMT0.5	0.5
BC-MMT1	1.0
BC-MMT2	2.0

**Table 3:** Experimental program of pure BC and BC-MMT biocomposite analysis.

Specimen	MMT Concentration (%)	Test Types		
		Physical/Barriers	Morphological	Thermal
BC	0.0	Water Activity Water Absorption Capacity Grammage Thickness Water Holding Capacity Water Release Rate	Scanning Electron Microscopy	Thermogravimetric Analysis
BC-MMT0.5	0.5			
BC-MMT1	1.0			
BC-MMT2	2.0			

**Table 4:** Comparison of reports of BC production by *G. hansenii* in various media.

Microorganism	Carbon Source	Supplementary Materials	Culture Time (Days)	Culture Method	Reference
G. *hansenii* ATCC 23769	Fructose	Glacial acetic acid	7	Static	[57]
G. *hansenii* ATCC 23769	Glucose	-	10	Static	[58]
G. *hansenii* ATCC 23769	Glucose	Galactose	7	_	[59]
G. *hansenii* ATCC 23769	Glucose	Cellulose nanofibril Nanoclay xGnP	9	Static/Agitated	[60]
G. *hansenii* ATCC 23769	Mannitol	D-(+)-glucose Dextrin	7	Static	[61]
G. *hansenii* ATCC 23769	Glucose	-	5	Static	[62]
G. *hansenii* ATCC 23769	Mannitol	-	14	Agitated	[63]
G. *hansenii* NCIM 2529	Sucrose	CaCl_2_	5	Agitated	[64]
G. *hansenii* UCP 1619	Glucose Acetylated glucose Molasses	Corn steep liquor	10	Static	[65]
G. *hansenii* CGMCC 3917	Glucose	Hydrolysate of waste beer yeast	10	Static	[66]

**Table 5:** Physical and barrier properties of flexible formulations of pure BC and BC-MMT composites. No significant difference between values with the same superscript letter ^(a,b,c)^ in a column (*p* > 0.05), according to Tukey’s test with 95% confidence.

Sample	Water activity	Grammage (g m^−2^)	Thickness (mm)	Water Holding Capacity (g_water_ g_sample_^−1^)
BC	0.479^b^ ± 0.002	0.047^c^ ± 0.005	81.250^c^ ± 11.242	87.729^a^ ± 4.032
BC-MMT0.5	0.529^a^ ± 0.002	0.113^b^ ± 0.016	133.667^b^ ± 15.462	46.023^b^ ± 3.497
BC-MMT1	0.519^a^ ± 0.006	0.153^ab^ ± 0.045	145.667^b^ ± 8.221	34.550b^c^ ± 3.930
BC-MMT2	0.516^a^ ± 0.002	0.209^a^ ± 0.054	162.333^a^ ± 2.571	29.514^c^ ± 7.165
